# Low Levels of Complement Factor H in the First Trimester of Pregnancy Are Associated with Spontaneous Preterm Birth

**DOI:** 10.3390/ijms251910549

**Published:** 2024-09-30

**Authors:** Carlos Hernán Becerra-Mojica, Eliana Mora-Guevara, Miguel Antonio Parra-Saavedra, Ruth Aralí Martínez-Vega, Luis Alfonso Díaz-Martínez, Bladimiro Rincón-Orozco

**Affiliations:** 1 School of Medicine, Universidad Industrial de Santander, Bucaramanga 680002, Colombia; elianadb1@hotmail.com (E.M.-G.); ladimar@uis.edu.co (L.A.D.-M.); 2 Centro de Atención Materno-Fetal INUTERO, Floridablanca 681004, Colombia; 3 Obstetrics and Gynecology Department, Universidad Libre, Barranquilla 080003, Colombia; miguelparra51@hotmail.com; 4 Escuela de Medicina, Universidad de Santander, Bucaramanga 680002, Colombia; rutharam@yahoo.com

**Keywords:** alternative complement pathway, biomarker, C3, Factor B, factor H and preterm birth

## Abstract

Preterm birth (PTB) remains a significant public health concern, and prediction is an important objective, particularly in the early stages of pregnancy. Many studies have relied on cervical characteristics in the mid-trimester, with limited results. It is therefore crucial to identify novel biomarkers to enhance the ability to identify women at risk. The complement pathway is implicated in the process of placentation, and recent proteomics studies have highlighted the potential roles of some complement proteins in the pathophysiology of PTB. To determine the association between the occurrence of spontaneous preterm birth (sPTB) and the concentration of complement C3, factor B, and factor H in the blood of pregnant women during the first trimester. This prospective cohort study included women with singleton pregnancies, both with and without a history of sPTB, from two health institutions in Bucaramanga, Colombia. The outcome was sPTB before 37 weeks. A blood sample was obtained between 11 + 0 to 13 + 6 weeks. ELISA immunoassay was performed to quantify the levels of C3, factor B, and factor H. A total of 355 patients were analyzed, with a rate of sPTB of 7.6% (27/355). The median plasma concentration for C3, factor B, and factor H were 488.3 μg/mL, 352.6 μg/mL, and 413.2 μg/mL, respectively. The median concentration of factor H was found to be significantly lower in patients who delivered preterm compared to patients who delivered at term (382 μg/mL vs. 415 μg/mL; *p* = 0.034). This study identified a significant association between low first-trimester levels of factor H and sPTB before 37 weeks. These results provide relevant information about a new possible early biomarker for sPTB. However, the results must be confirmed in different settings, and the predictive value must be examined

## 1. Introduction

Preterm birth (PTB), defined as childbirth occurring before 37 weeks of gestation, represents a significant global health challenge [1]. According to WHO estimates, approximately 15 million PTB cases occur annually [2]. Despite the implementation of research and public health programmes, the PTB rate has remained unchanged over the past decades [3]. Furthermore, in some regions, the rates have even increased [4]. PTB remains the leading cause of neonatal and under-five mortality, contributing to the deaths of approximately one million neonates annually due to PTB-related complications [5]. A history of previous PTB has been demonstrated as a risk factor worldwide [6]. Nevertheless, 80% of pregnant women who deliver preterm are in their first pregnancy, or the previous pregnancy ended at term. Another risk factor is a mid-trimester cervical length of less than 25 mm. This biomarker can identify 28% of patients who will deliver before 37 weeks [7]. Through a tailored approach, the detection rate of spontaneous preterm birth (sPTB) before 37 weeks using cervical length can be substantially enhanced, reaching as high as 50% [8].

The administration of vaginal progesterone to patients with a cervical length of less than 25 mm in the middle trimester has been demonstrated to reduce the risk of PTB by 38% [9]. This management protocol is cost-effective in different populations [10,11]. However, there are instances where patients with a cervical length greater than 25 mm experience premature delivery, and patients with a short cervix also experience premature delivery despite the use of progesterone. These findings underscore the necessity for further investigation into the development of novel biomarkers that can enhance the predictive value of cervical measurements, particularly at the earliest stages of the disease.

A recent systematic review aimed at identifying serum biomarkers for the prediction of PTB concluded that, at the time of the review, no single or combined biomarker could be identified that allowed for such prediction [12]. This is likely due to the heterogeneous nature of PTB and the incomplete understanding of the mechanisms that drive this pathological process [13]. The complement pathway has been studied in pregnancy not only as a participant in the normal process of placentation but also in the pathophysiology of preterm birth [14,15]; many components have been found to be elevated in pregnant women who deliver preterm, namely Bb [16], C3a [17], and C5b9 [18]. A mechanistic model of complement activation arising from the vaginal microbiota composition has also been postulated [19]. Discovery methodologies have contributed to the expansion of knowledge regarding the biological systems involved in preterm birth; two recent proteomic studies using different techniques identified proteins differentially expressed in pregnant women who experienced a PTB; some of them belong to the alternative pathway of the complement [20,21]. Based on the results of these studies, we selected three candidates for evaluation: C3 as the initiator, factor B as the cofactor, and factor H as the main controller of the alternative complement pathway. The aim of the study was to establish whether the concentrations of the three proteins, C3, factor B, and factor H, differed between patients who delivered before 37 weeks and those who delivered at term.

## 2. Results

### 2.1. Description of the Cohort and Characteristics of the Study Population

**Figure 1:**
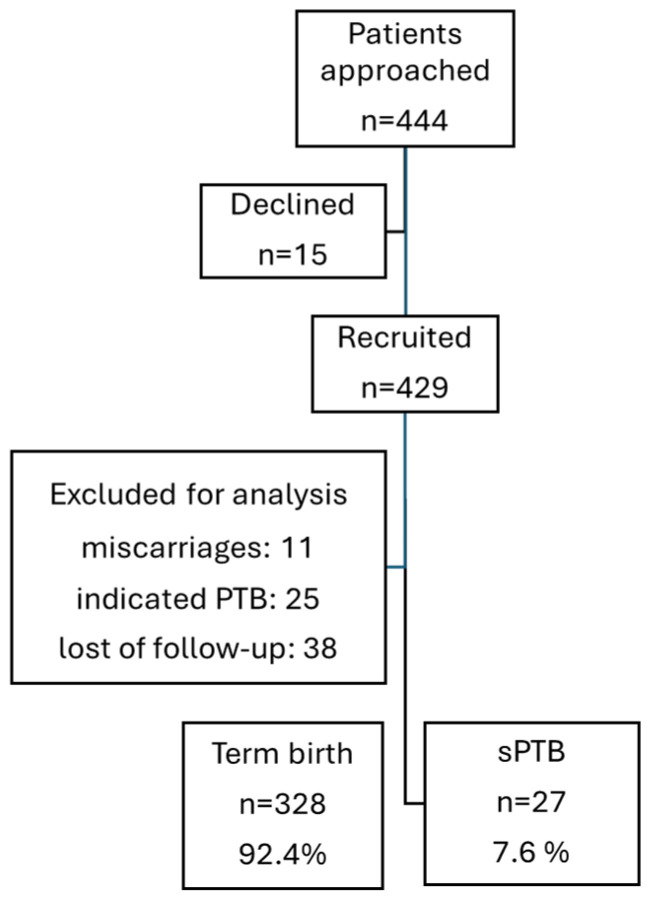
Patient flowchart.

Among the 429 pregnant women initially recruited, 74 were excluded due to miscarriage 11 (15%), indicated preterm birth 25 (34%), and incomplete perinatal outcomes 38 (51%) (Figure 1). The causes of the indicated preterm birth were preeclampsia (12), cholestasis (3), abruptio placentae (2), intrauterine growth restriction (IUGR) (4), no satisfactory fetal status (3), and intrauterine fetal death (IUFD) (1).

For the 355 women remaining for analysis, the rate of sPTB < 37 weeks was 7.6% (27/355). The median maternal age was 28 years, while 2% (7/355) of the population had a history of PTB. Women who delivered before 37 weeks of gestation exhibited a higher prevalence of a history of PTB (14.8% vs. 0.9%, *p* < 0.001). In addition, a lower cervical length (34 mm vs. 35 mm, *p* = 0.108) was observed in patients with sPTB, but these differences were not significant. No significant differences were observed in body mass index (25.8 vs. 25.1; *p* = 0.934), smoking habits (11.1% vs. 11.9%; *p* = 0.908), or the median of the baseline gestational age (Table 1). Supplementary Table S1 contains the clinical and demographic characteristics of the entire cohort. The median gestational age at delivery was 39.0 weeks (IQR 38–39.5) in patients who delivered at term, while it was 34.2 weeks (IQR 34.0–36.3) in patients who delivered preterm.

### 2.2. First Trimester Levels of Complement C3, Factor B, and Factor H

The plasma levels of the three complement proteins were established. The median concentration level for C3 was 488.3 µg/mL (IQR 385.2–684.2), the median level for factor B was 352.6 µg/mL (IQR 264.2–475.4), and the median level for factor H was 413.2 µg/mL (IQR 320.0–484.6).

### 2.3. Association between Complement Proteins and sPTB before 37 Weeks

**Figure 2:**
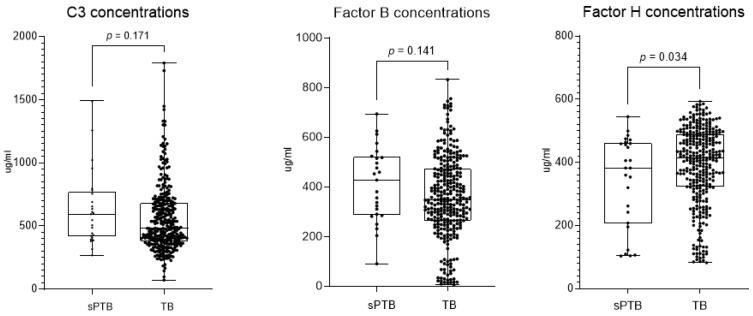
Complement protein levels by preterm or term birth.

A statistically significant difference was identified in complement factor H concentration between women who delivered preterm and those who delivered at term. The concentration was 382 µg/mL in the former group, compared to 415 µg/mL in the latter (*p* = 0.034). Concentrations of complement C3 and factor B were observed to be higher in pregnant women who delivered preterm compared to women who delivered at term; however, the differences were not statistically significant, with *p*-values of 0.171 and 0.141, respectively (Table 1 and Figure 2).

Furthermore, the association between complement proteins and sPTB was investigated using percentiles 5, 10, and 25. Patients with factor H levels below percentile 5 (117.5 µg/mL) exhibited almost a fourfold probability of sPTB (OR 3.90). Also, percentile 10 (200.8 µg/mL) demonstrated almost a threefold probability (OR 2.95). The percentile 25 (320.0 µg/mL) was not associated with the outcome (Table 2). This result remained consistent even after adjusting for maternal age and history of preterm birth. Evaluation of percentiles 75, 90, and 95 for C3 and factor B did not yield any significant findings.

## 3. Discussion

### 3.1. Main Finding

This is the first prospective cohort study to demonstrate an association between diminished plasma factor H concentrations and sPTB before 37 weeks gestation. The differential concentrations of the complement proteins studied suggest a pathological higher activity of the alternative complement pathway from the first trimester of the pregnancy in patients who delivered before 37 weeks. Higher levels of C3 and factor B indicate an increased complement activity, while the significantly lower level of factor H probably reflects an intense response aimed at controlling exaggerated activity at this point.

### 3.2. What We Know and What Is New

Complement factor H plays a pivotal role in regulating the alternative pathway. It is a protein comprising 20 repeating units of 60 amino acids, known as short consensus repeat. This regulatory protein is encoded on chromosome 1 within a cluster of genes designated as regulators of complement activation (RCA). The RCA cluster contains more than sixty genes, all encoding members of the factor H family [22]. The regulatory function of factor H is exerted at various levels within the alternative pathway cascade. One of its functions is to act as a cofactor for Factor I (FI)-mediated proteolysis of C3b into iC3b, which is unable to further propagate pathway activation. Furthermore, factor H also competes with factor B (FB) to inhibit the formation of the C3(H_2_O)B fluid phase tick-over complex. Additionally, factor H promotes the decay of existing C3bBb complexes (i.e., the C3-convertase), as well as the C4bC2aC3b and C3bBbC3b complexes (i.e., the C5 convertases) [22].

There is considerable variability in the plasma levels of factor H in humans. It has been documented that plasma concentrations increase with age, while lower levels have been observed among smokers. However, it is considered that the greater variability is dependent on the composition of the genetic determinants of the factor H factor coding [23]. Information about the plasma levels of factor H in pregnant women is limited. Despite the lack of standardisation in the quantification of factor H, studies in this regard commonly show that normal pregnant women have higher plasma levels than non-pregnant women [24,25,26].

Although the levels of complement factor H during pregnancy vary in the reviewed studies, these variations could be explained by the different protein quantification techniques. Despite these differences, the dynamics derived from studies that obtained samples at different stages of pregnancy show an increase that is evident from week 6 of pregnancy, with maximum values at the end of the first trimester and the beginning of the second trimester. Subsequently, they exhibit a plateau [27,28], with a decrease in values towards normality during the postpartum period [27].

In the context of factor H behaviour in patients with obstetric pathology, some studies have demonstrated low plasma levels in patients who developed preeclampsia [29], particularly in early-onset cases [26,30]. For the case of PTB, we did not find prospective clinical studies oriented to establish an association with factor H at any time of the pregnancy. In the Beernike study, the authors found differentially expressed factor H in the discovery phase, and the ELISA quantification showed low concentrations of factor H in preterm birth patients; however, no statistical significance was found [31].

Considering that an uncontrolled inflammatory state represents a biological basis of a subset of patients who deliver before 37 weeks, some complement components have been studied, with varying degrees of association observed. For instance, Lynch et al. identified a significant correlation between elevated complement fragment Bb before 20 weeks of gestation and PTB before 34 weeks. Patients with Bb fragment levels in the superior quartile exhibited a fourfold increased risk of PTB [16]. In a subsequent study from the Lynch group, another complement fragment, anaphylatoxin C3a, was evaluated. This study identified an association between sPTB before 37 weeks and higher levels of C3a in early pregnancy [17].

The results of the present study are consistent with the hypothesis proposed by the authors mentioned, namely that an uncontrolled inflammatory environment is present in the first trimester of pregnancy in patients presenting adverse outcomes, including sPTB. The findings indicate that pregnant women with PTB exhibit higher values of C3 and factor B from the first trimester. Although these differences were not statistically significant, these results suggest that in this group of patients, there is greater activity in the alternative complement pathway, which is accompanied by greater activity of factor H to control this pathological process. Our hypothesis is that the markedly reduced factor H concentrations indicate system-wide exhaustion resulting from the high consumption at the different action levels in the complement cascade.

This is the most plausible explanation, given that previous studies have shown that other downstream proteins are not increased, indicating that the process is controlled to the extent possible so that it does not exceed the activation of the C3 convertase and C5 convertases [28]. An alternative hypothesis is that there is a low production of factor H. This hypothesis should be explored by seeking genetic mutations in chromosome 1. However, if this were the case, there would be expected evidence of increased activity in the complement system downstream from the implantation process, which has not yet been demonstrated. Further studies are required to test this hypothesis.

This study provides relevant information about the abnormal processes occurring in the first trimester of pregnancy in a woman who deliver preterm and highlights the characteristics of complement factor H as a potential biomarker for identifying a pregnant woman at risk of sPTB. However, the predictive capacity must be validated in other larger cohorts. The findings indicate the possibility of enhancing the prediction of sPTB and investigating novel therapeutic targets within the complement pathway. This could offer a chance to modify the disease trajectory at an early stage, although this hypothesis requires further substantiation.

### 3.3. Strengths and Limitations of the Study

This study has several notable strengths. Firstly, it is prospective in nature, which allows for the observation of outcomes over time. Secondly, gestational age was determined with precision based on the ultrasound CRL obtained in the first trimester, which is a crucial aspect in perinatal research. Thirdly, the same professional team conducted all the assays, ensuring consistency and reliability in the data collection process. In addition, the ELISA kit used has shown reliable characteristics for detecting the main member of the factor H family [21]. The rigor of the assays was confirmed by the R2, and the inter- and intraassay CV were below the recommended limits. This study contributes to the knowledge base from a low-middle-income country. However, the findings may not be generalizable to other populations, as the study population consisted of women from a Colombian region. Additionally, the alternative complement pathway and complement behaviour during pregnancy were not explored, which could have provided further insights.

## 4. Materials and Methods

### 4.1. Study Design and Participants

The study population consisted of a prospective cohort of singleton pregnant women between 11 + 0 and 13 + 6 weeks of gestation, recruited between September 2020 and January 2023. Participants were recruited from the maternal–fetal units of two health institutions in the Metropolitan area of Bucaramanga, Colombia: Centro de Atención Maternal-Fetal INUTERO and Centro de Atención Materno-Fetal Colombia. The inclusion criteria were pregnant women between 11 + 0 and 13 + 6 weeks with a singleton pregnancy, including previous sPTB and nulliparous women. Patients with chronic and autoimmune diseases were not included. Women were excluded from the study if they had a history of cervical surgery or Mullerian anomalies, had experienced PTB due to fetal or maternal indications (non-spontaneous), or had experienced a pregnancy that had ended before 22 weeks of gestation. The Committee on Ethics and Research from the Universidad Industrial de Santander and the participating canters approved the study. All pregnant women provided written informed consent for participation in this study.

### 4.2. Recruitment and Baseline Evaluation

Women were invited to participate in the 11 + 0 to 13 + 6-week screening period. Data, including age, obstetric history, history of PTB, and cervical procedures, were obtained from pregnant women using a standardized survey during enrolment. Anthropometric data were obtained before the ultrasound evaluation to determine typical risk calculations for chromosomal abnormalities and risk for preeclampsia. In addition, blood was collected from the forearm in a tube with EDTA K2 anticoagulant. The sample was centrifugated at 3000 rpm for 10 min; the plasma was collected and stored at −70 °C until the laboratory analysis was performed.

### 4.3. Ultrasound Evaluation

The examinations were conducted by highly skilled maternal–fetal specialists, each with over five years of experience in 11 + 0 to 13 + 6 ultrasound screening scans. The ultrasound evaluation involved assigning gestational age based on crown-to-rump length (CRL), biometric measurements, and cervical length measurement, utilizing a 4–9 MHz endocavitary probe (Voluson E6, S8 General Electric, Milwaukee, MI, USA). The physicians were blinded to the complement concentrations, and medical decisions were not made based on these results.

### 4.4. ELISA Immunoassay Test

Three components of the alternative complement pathway were evaluated, including C3, factor B, and factor H. We used the enzyme-linked immunosorbent assay (ELISA) Abcam kits; (ab108823—Complement C3 Human ELISA Kit, Cambridge, UK, ab137973, Human Factor B ELISA Kit, Cambridge, UK, and ab252359 Human Complement Factor H SimpleStep ELISA^®^ Kit, Cambridge, UK). The assays were performed following the manufacturer’s instructions. We used the colorimetric technique to establish the levels of the complement proteins in the plate reader CLARIOStarPlus BMG LABTECH (Ortenberg, Germany). Two researchers performed all the laboratory assays along with the study to maintain the stability of the technique. The standard curve was determined by regression analyses using a four-parameter logistic curve fit. For each experiment, the reference curves checked with R2 were between 0.992 and 0.999. Additionally, we evaluated at least ten random samples per well plate and samples on different plates to test the variability coefficient inter-assay and intra-assay. The intra-assay CV% was 7%, while the inter-assay CV% was 12%.

### 4.5. Follow-Up Evaluation

After the initial evaluation, participants continued their scheduled medical controls following institutional guidelines. The outcome was the occurrence of sPTB, defined as childbirth before 37 weeks of gestation.

The research team contacted pregnant women to monitor the pregnancy until delivery. The date of delivery, the delivery route, the delivery characteristics, and whether it was spontaneous or indicated by fetal or maternal conditions were recorded. The reason for the delivery was also recorded. Data were confirmed from the participant’s medical records.

### 4.6. Data Collection

Clinical data were stored in a password-protected, web-based electronic database, REDCap, with de-identification capability to protect patient information. The complement concentrations were added to the database as the laboratory tests were performed according to the assigned code.

### 4.7. Statistical Analysis

For describing quantitative variables, the median and interquartile range (IQR) were utilized because the data did not have a normal distribution. Categorical data were expressed as proportions and percentages. C3, factor B, and factor H levels were compared between patients who delivered preterm and those who delivered at term using the Mann–Whitney U test, and qualitative variables were compared using the Chi2 test. The association between C3, factor B, factor H, and spontaneous PTB concentrations was evaluated through logistic regression analysis (StataCorp. 2020, Stata Statistical Software: Release 16. College Station, TX, USA). The figures for protein concentrations were created using GraphPad Prism 10.

## 5. Conclusions

The complement alternative pathway is highly active from the beginning of pregnancy. This activity is tightly regulated, and disruption of this delicate balance can lead to adverse pregnancy complications, including preterm birth. In this study, significantly low levels of factor H were identified in the first-trimester plasma of pregnant women who delivered before 37 weeks. The findings contribute to the understanding of the pathophysiological processes associated with sPTB and suggest that factor H may serve as a potential early biomarker for sPTB.

## Figures and Tables

**Table 1:** Baseline characteristics of the including population by type of delivery.

Characteristics	Delivered at Term *n* = 328	sPTB < 37 Weeks *n* = 27	*p*-Value
Maternal age years *	28 (24–32)	26 (23–29)	0.118
Preterm birth history	3 (0.91%)	4 (14.8%)	<0.001
Smoking history	39 (11.9%)	3 (11.1%)	0.928
Body mass index *	25.1 (22.6–28.1)	25.8 (21.8–29.3)	0.934
Gestational age weeks *	13.1 (12.5–13.5)	13.0 (12.4–13.4)	0.329
Cervical length mm *	35 (33–37)	34 (32–36)	0.108
C3 concentration μg/mL	481 (379–679)	597 (421–755)	0.171
Factor B concentration μg/mL	348 (263–472)	427 (289–517)	0.141
Factor H concentration μg/mL	415 (323–489)	382 (208–459)	0.034
Socioeconomic strata			
1, 2 **^†^**	176 (53.6%)	16 (59.2%)	
3, 4, 5, 6	152 (46.3%)	11 (40.7%)	0.634
Marital status			
Live with partner	281 (85.7%)	27 (100%)	0.480
Single	47 (14.3%)	0	---
Local infection			
Negative	99 (30.2%)	8 (29.6%)	
Vaginal	170 (51.8%)	15 (55.5%)	0.419
Urinary tract infection	59 (17.9%)	4 (14.8%)	0.801
Residence place **			
Metropolitan area	280 (85.4%)	20 (68.8%)	
Outside	48 (14.6%)	7 (31.2%)	0.217
Nationality **			
Colombian	321 (97.8%)	26 (95.1%)	
Venezuelan	7 (2.2%)	1 (4.9%)	0.991

* Median (IQR); sPTB: spontaneous preterm birth; CCI: cervical consistency index; GA: gestational age. ** Some patients did not answer the question. ^†^ Category 1, 2 represent low economic resources.

**Table 2:** Association of the factor H concentrations by percentiles and preterm birth before 37 weeks.

Percentile/Value	OR (95% CI)	*p*	OR a * (95% CI)	*p*
C3				
>75 (684.20)	1.36 (0.57–3.26)	0.482	1.34 (0.55–3.22)	0.519
>90 (908.50)	1.67 (0.54–5.15)	0.372	1.71 (0.54–5.15)	0.357
>95 (550.16)	0.79 (0.10–6.26)	0.829	0.83 (0.10–6.68)	0.864
Factor B				
>75 (484.63)	2.27 (0.98–5.27)	0.056	2.50 (1.05–5.90)	0.037
>90 (528.58)	1.85 (0.59–5.75)	0.286	1.86 (0.58–5.87)	0.292
>95 (550.16)	0.79 (0.10–6.26)	0.829	0.83 (0.10–6.68)	0.864
Factor H				
<5 (117.55)	3.90 (1.19–12.81)	0.025	3.37 (1.00–11.29)	0.049
<10 (200.78)	2.95 (1.10–7.88)	0.031	2.83 (1.04–7.71)	0.041
<25 (320.02)	1.92 (0.84–4.36)	0.120	1.86 (0.81–4.28)	0.142

* Adjusted by maternal age and PTB history.

## Data Availability

The data that support the findings of this study are available on re-quest from the corresponding author.

## Supplementary Materials

The supporting information can be downloaded at: https://www.mdpi.com/article/10.3390/ijms251910549/s1.
