# Supplementary material for: Low Levels of Complement Factor H in the First Trimester of Pregnancy Are Associated with Spontaneous Preterm Birth
Source: Int J Mol Sci. 2024 Sep 30;25(19):10549. doi: 10.3390/ijms251910549 (PMC11476428; doi:10.3390/ijms251910549)
Supplement: Supplementary file 1 [file ijms-25-10549-s001.zip › ijms-3188736-supplementary.pdf]

Table S1. Demographic and clinical characteristics of the entire cohort (n=355)

**Characteristic**	**n**	**%**
**Age (years)***	28 (24-32)	
Recruitment center		
INUTERO	311	87.6
Maternofetal Colombia	44	12.4
Marital status		
Single	47	13.2
Live with a partner	308	86.8
Place of residency		
Metropolitan area	300	84.5
Outside	55	15.5
Health care		
Subsidized	78	21.9
Contributive	268	75.6
Special	9	2.5
Labor		
Employee	179	50.4
Freelance	59	16.6
Home	98	27.6
Student	18	5.1
Unemployed	1	0.3
Pregnancy number		
0	140	39.6
1	138	39.0
2	49	13.9
3 +	26	7.4
Preterm birth history		
Yes	7	2.0
No	177	49.9
First pregnancy	171	48.1
Smoking history		
Yes	42	11.8
No	313	88.2
Body Mass Index		
Underweight	13	3.7
Normal	162	45.6
Overweight	118	33.2
Obesity	62	17.5
Gestational age at recruitment*	13.2 (12.5-13.6)	

Cervical length (mm)*	34 (31-36)
Gestational age at delivery*	38.6 (37.6-39.4)
C3 concentration (ug/ml)*	488.3 (385.2-684.2)
Factor B concentration (ug/ml)*	352.6 (264.2-475.4)
Factor H concentration (ug/ml)*	413.2 (320.0-484.6)
Gestational age at delivery*	38.6 (37.6-39.4)

*Median (IQR)
